# Mendelian randomization analysis to assess a causal effect of haptoglobin on macroangiopathy in Chinese type 2 diabetes patients

**DOI:** 10.1186/s12933-018-0662-7

**Published:** 2018-01-16

**Authors:** Shiyun Wang, Jie Wang, Rong Zhang, Tao Wang, Dandan Yan, Zhen He, Feng Jiang, Cheng Hu, Weiping Jia

**Affiliations:** 1 Shanghai Diabetes Institute, Shanghai Key Laboratory of Diabetes Mellitus, Shanghai Clinical Center for Diabetes, Shanghai Jiao Tong University Affiliated Sixth People’s Hospital, Shanghai, 200233 People’s Republic of China; 2 Institute for Metabolic Disease, Fengxian Central Hospital Affiliated to Southern Medical University, 6600 Nanfeng Road, Shanghai, 201499 People’s Republic of China

**Keywords:** Haptoglobin, Mendelian randomization, Macroangiopathy, Type 2 diabetes

## Abstract

**Background:**

Haptoglobin (Hp) functions as an antioxidant by binding with haemoglobin. We investigated whether serum Hp has a causal effect on macroangiopathy via Mendelian randomization (MR) analysis with common variants of the Hp gene in Chinese patients with type 2 diabetes.

**Methods:**

A total of 5687 type 2 diabetes patients were recruited and genotyped for the Hp gene. Clinical features and vascular imaging tests were applied to diagnose macroangiopathy. The association between common Hp genotypes and macroangiopathy was analyzed in the whole population. Serum Hp levels were measured by enzyme-linked immunosorbent assay in a subset of 935 patients. We individually analyzed the correlations among Hp levels, Hp genotypes and macroangiopathy. Further, 8-hydroxy-2′-deoxyguanosine (8-OHdG), an oxidative marker of DNA damage, was examined to evaluate the levels of oxidative stress.

**Results:**

Common Hp genotypes were correlated with macroangiopathy (OR = 1.140 [95% CI 1.005–1.293], *P* = 0.0410 for the Hp 1 allele). Serum Hp levels were associated with both common Hp genotypes (*P* = 3.55 × 10^−31^) and macroangiopathy (OR = 2.123 [95% CI 1.098–4.102], *P* = 0.0252) in the subset of 935 patients. In the MR analysis, the directional trends of the observed and predicted relationships between common Hp genotypes and macroangiopathy were the same (OR 1.357 and 1.130, respectively). Furthermore, common Hp genotypes and Hp levels were associated with serum 8-OHdG levels (*P* = 0.0001 and 0.0084, respectively).

**Conclusions:**

Our study provides evidence for a causal relationship between serum Hp levels and macroangiopathy in Chinese type 2 diabetes patients by MR analysis.

**Electronic supplementary material:**

The online version of this article (10.1186/s12933-018-0662-7) contains supplementary material, which is available to authorized users.

## Background

Type 2 diabetes mellitus has become one of the most challenging public health problems worldwide. In China, it is estimated that more than 100 million individuals are affected by diabetes [1, 2]. Diabetes can lead to serious pathological damage of numerous organs and tissues, among which macroangiopathies are the major causes of morbidity and mortality [3]. Oxidative stress, which results from the overproduction of reactive oxygen species, is closely associated with the pathogenesis of diabetes. Long-term exposure to oxidative stress causes chronic inflammation, endothelial dysfunction and fibrosis, leading to the formation of various macroangiopathies in diabetes patients [4]. Therefore, natural antioxidative stress agents in the human body are crucial in the development of diabetic macroangiopathies.

Haptoglobin (Hp) is a haemoglobin (Hb)-binding protein that was detected in 1938 [5]. Free Hb can lead to the accumulation of hydroxyl radicals and damage to vascular endothelial cells via the Fenton reaction [6]. As an antioxidant, Hp is notable for its biological ability to scavenge free Hb and prevent oxidative tissue damage [7]. This protein, which is mainly synthesized by hepatocytes, is encoded by the Hp gene. Two common alleles (Hp 1 and Hp 2) exist due to copy number variation of a tandem two-exon segment, yielding three common phenotypes: Hp 1-1, Hp 1-2 and Hp 2-2 [8]. Additionally, an allelic deletion larger than 20 kb results in a less common allele, Hp del, which is associated with hypohaptoglobinemia [9].

Common variants of the Hp gene are major genetic factors influencing serum Hp level in both the general population and in patients with various diseases, such as thalassemia, polycystic ovary syndrome and cardiovascular diseases (CVDs) [10–15]. Furthermore, common variants of the Hp gene were reported as an independent risk factor for CVDs in patients with diabetes compared with non-diabetic patients [16, 17]. A higher level of serum Hp was not only observed in experimental diabetic rats during the early stage of diabetes, but was also found to be correlated with CVDs in various epidemiological studies [18–20]. However, whether serum Hp has a causal effect on macroangiopathy in type 2 diabetes remains uncertain due to the influences of various confounding factors and a lack of clarity regarding reverse causality.

Mendelian randomization (MR) analysis, which is highly similar to a randomized controlled trial (RCT), can shed light on a causal inference from an observational study. Based on Mendel’s laws, genetic variants are inherited randomly and remain constant over time. Viewed as a surrogate of the related exposure and as an instrumental variable (IV), genetic variants are considered free from potential confounding factors and reverse causation [21]. Therefore, several MR studies have investigated the causal role of multiple biomarkers, including various lipid and inflammation traits, as risk factors for CVDs and diabetes [22].

In this study, we aimed to determine whether serum Hp had a causal effect on macroangiopathy in Chinese type 2 diabetes patients via MR analysis. According to Mendel’s laws, the major criterion that a genetic variant must meet before it can be considered as a valid IV is that there exists robust evidence of a true association between the genetic variant and exposure of interest [21]. Based on the well-known relationship between common Hp genotypes and serum Hp levels evidenced from previous studies, common variants of the Hp gene were selected as an IV in the current study. First, the association between the common Hp genotypes and macroangiopathy was tested in 5687 patients. Then, 935 subjects from the total population were selected to measure serum Hp levels and to verify the relationship between Hp levels and common Hp genotypes. Second, we determined whether serum Hp levels were correlated with macroangiopathy. Finally, by comparing the observed and predicted associations between common variants of the Hp gene and macroangiopathy, we evaluated whether Hp had a causal effect on macroangiopathy. In addition, serum 8-hydroxy-2′-deoxyguanosine (8-OHdG), an oxidant of deoxyguanosine, was selected to detect the extent of DNA damage and evaluate the oxidative stress levels of patients.

## Methods

### Ethical approval

Ethical approval was granted by the Institutional Review Board of Shanghai Jiao Tong University Affiliated Sixth People’s Hospital according to Helsinki Declaration II. All subjects provided written informed consent to participate.

### Participants

We recruited 5687 Chinese patients diagnosed with type 2 diabetes by the 1999 World Health Organization criteria from the Shanghai Diabetes Institute Inpatient Database. The study included 3078 males and 2609 females with an average age of 59.64 ± 12.25 years and an average body mass index (BMI) of 24.78 ± 4.38. Patients with type 1 diabetes, haemolytic disease, cancer, pregnancy, cardiac failure or drug or alcohol addiction were excluded from this study. In addition, patients known to have any severe infection (such as recently diagnosed diabetic foot infection) that might influence serum Hp levels or 8-OHdG levels were excluded from the study.

### Clinical measurements

The anthropometric features of all 5687 patients, such as age, sex, height and weight, were recorded. BMI was calculated as weight (kg)/height^2^ (m^2^). Blood pressure was measured by an experienced physician using a mercury sphygmomanometer. Fasting venous blood samples were obtained, and serum was aliquoted into plastic tubes and stored at − 80 °C until analysis. HbA1c levels were determined using high-performance liquid chromatography with a Bio-Rad Variant II Hb testing system (Bio-Rad Laboratories, Hercules, CA). Serum concentrations of lipid profiles, including total cholesterol (TC), triglycerides (TG), high-density lipoprotein cholesterol (HDL-C) and low-density lipoprotein cholesterol (LDL-C), indexes of liver function, including serum alanine aminotransferase (ALT) and aspartate aminotransferase (AST) [23], and serum albumin concentration to evaluate nutritional status [24], were assayed on a Hitachi 7600-020 automated biochemical analyser (Hitachi, Tokyo, Japan). The serum high sensitive C-reactive protein (hsCRP) level was detected by using CardioPhase* hs-CRP reagent in a particle-enhanced immunoturbidimetric assay (Dade Behring, Newark, USA).

### Serum Hp measurement

A subset of 935 subjects (500/435 patients with/without macroangiopathy, respectively) from the total population with sufficient frozen serum samples were selected to determine serum Hp levels via enzyme-linked immunosorbent assay (ELISA) using the Human Haptoglobin Quantikine ELISA kit (catalogue #DHAPG0; R&D Systems, Inc., Minneapolis, USA) according to the manufacturer’s instructions.

### Serum 8-OHdG measurement

8-OHdG levels were measured in the 935 subjects using the Highly Sensitive 8-OHdG Check ELISA kit (code KOG-HS10E; JaICA, Fukuroi City, Japan). To accurately detect 8-OHdG in serum, which contains high-molecular weight substances, such as proteins, proteins were removed from the serum sample [25]. According to the manufacturer’s instructions regarding sample pretreatment, an ultrafilter Microcon YM-10 (catalogue #42407; Millipore Corporation, Temecula, USA) with a molecular weight cut-off of 10 kDa was used to filter the serum samples. A total of 150–200 µL of filtrate was obtained, and the content of serum 8-OHdG was determined by ELISA.

### Diagnosis of macroangiopathy in diabetes

A vascular ultrasound was used to examine the bilateral carotid and lower limb arteries. Carotid arteries were examined bilaterally at the following four locations: the common carotid arteries, the bifurcation, the internal and the external carotid arteries. The lower limb arteries were tested at the following seven locations: the common femoral artery, the superficial and the profunda femoral arteries, the popliteal artery, the anterior and the posterior tibial arteries, and the peroneal artery [26]. Information on the intima-media thickness, atherosclerotic plaques and stenosis was recorded at each location. An atherosclerotic plaque was defined as a focal protrusion into the arterial lumen of 0.5 mm or 50% greater than the surrounding intima-media thickness value [27]. A significant artery stenosis was evaluated by conventional Doppler ultrasound criteria, as reported [28]. Carotid and lower limb atheroscleroses were diagnosed as the presence of carotid and lower limb plaques or significant stenosis in any of the abovementioned arterial segments, respectively. Computed tomography and/or magnetic resonance imaging were utilized to obtain evidence of cerebrovascular disease. Myocardial infarction was diagnosed using the international guidelines for symptoms of clinical ischaemic, electrocardiographic and biochemical markers of myocardial necrosis changes or coronary angioplasty history. Patients with a history of any of the following disorders were diagnosed with macroangiopathy: carotid atherosclerosis, lower limb arteriosclerosis, myocardial infarction, stroke or intracerebral haemorrhage.

### Hp genotyping

All 5687 patients were genotyped using TaqMan assays via a 7900HT Fast Real-Time PCR System (Applied Biosystems, Foster City, CA) with modifications to the method previously described by Soejima et al. [29]. The 5 μL PCR reaction contained 20 ng of genomic DNA, 2.5 μL of TaqMan^®^ Genotyping Master Mix (Applied Biosystems, Foster City, CA), 0.4 μL of Ampli*Taq* Gold (Applied Biosystems, Foster City, CA), and TaqMan primers and probes as follows: Hp 2-F and Hp 2-R primers (900 nmol/L) and a VIC-labelled probe (250 nmol/L) to detect Hp 2, Hp 5′-F and Hp 5′-R primers (500 nmol/L) and a ABY-labelled probe (200 nmol/L) to detect the Hp 5′ region, and Hp del-F and Hp del-R primers (900 nmol/L) and a FAM-labelled probe (250 nmol/L) to detect the Hp del. The PCR temperature profile was 95 °C for 10 min followed by 40 cycles of denaturation for 15 s at 95 °C and annealing and extension for 1 min at 60 °C. All primer and probe sequences and the calculation method to determine the Hp 2/Hp 5′ ratio were the same as previously described [29, 30].

### Statistical analyses

Statistical analyses of the quantitative characteristics were performed using SAS for Windows (version 9.2; SAS Institute, Cary, NC, USA). Normality tests were performed, and any skewed quantitative trait data were logarithmically transformed. Data are shown as n or the mean ± standard deviation or median (interquartile range). The t test and χ^2^ test were performed to analyze between-group differences for normally distributed quantitative and categorical variables, respectively. Correlations between the common Hp genotypes and clinical traits were analyzed by multiple linear regression. Our total samples had > 80% power to detect an estimated effect size (odds ratio ~ 1.2) of common variants of the Hp gene in influencing diabetic macrovascular diseases with a minor allele frequency of 0.25 and a level of significance of 0.05, as determined by the Quanto 1.2 software. A sample size of 500 patients with macroangiopathy and 435 subjects without macroangiopathy had > 80% power to detect a between-group mean difference in serum Hp levels (~ 10 mg/dL) based on a two-sided t test with a significance level of 0.05, as determined by the G*Power 3.1 software.

The MR analysis was conducted as previously described by using an IV [31]. Genes associated with exposure act as an IV and help in detecting a causal relationship between exposure and disease. In this research, common variants of the Hp gene (Hp 1-1, Hp 1-2 and Hp 2-2) were selected as an IV. The HW equilibrium calculator (http://www.oege.org/software/hwe-mr-calc.shtml) was used as previously described to test Hardy–Weinberg equilibrium for common Hp genotypes [32]. Correlations between common Hp genotypes and serum Hp levels were analyzed by multiple linear regression, and β_1_ values are presented. The association between Hp level and macroangiopathy was tested using multivariable logistic regression analysis, and the odds ratio (OR_2_) values with 95% confidence interval (CI) are presented. The observed association between common Hp genotype and macroangiopathy was analyzed under an additive model using multivariable logistic regression analysis, and OR_3_ values with 95% CI are presented. Combining the associations among common Hp genotypes, serum Hp levels and macroangiopathy, the predicted association between common Hp genotypes and macroangiopathy (OR_4_) was calculated by OR_4_ = exp(β_1_ × log_e_ OR_2_) and compared with the observed value (OR_3_) to verify the causal relationship. Two-tailed significance was set at *P* < 0.05.

## Results

### General characteristics

The patient characteristics grouped by Hp genotype are presented in Table 1. Of the participants, 5238 individuals carried at least one of the two major alleles (Hp 1 and Hp 2), and 449 subjects carried the Hp del allele. Among the patients without the Hp del allele (n = 5238), the common Hp genotypes were distributed as follows: Hp 1-1, 7.20% (n = 377); Hp 1-2, 38.70% (n = 2027); and Hp 2-2, 54.10% (n = 2834). These genotypes were in Hardy–Weinberg equilibrium overall (*P* = 0.5770). LDL-C and TC levels differed among the different common Hp genotypes. After adjusting for age, sex, BMI and blood pressure, common Hp genotypes were significantly associated with LDL-C (*P* < 0.0001) and TC (*P* < 0.0001) (see Additional file 1: Table S1). The prevalence of macroangiopathy was higher among patients with the Hp 1 allele (78.51% vs 72.57% vs 72.41% for Hp 1-1 vs Hp 1-2 vs Hp 2-2, respectively). After adjusting for age, sex, BMI, blood pressure, duration of diabetes, HbA1c, LDL-C and TC, common variants of the Hp gene exhibited a significant association with macroangiopathies (OR = 1.140 [95% CI 1.005–1.293], *P* = 0.0410 for the Hp 1 allele). Furthermore, diabetic macroangiopathies were divided into four classes (cerebral, carotid, coronary and lower limb atherosclerosis) to investigate the relationships between common variants of the Hp gene and each atherosclerosis (see Additional file 1: Table S2). After adjusting for confounding factors, we found that the common variants of the Hp gene were significantly associated with carotid atherosclerosis (OR = 1.183 [95% CI 1.027–1.363], *P* = 0.0202 for the Hp 1 allele).

**Table 1:** Clinical characteristics of all the patients by Hp genotype

Variable	Hp 1-1	Hp 1-2	Hp 2-2	Hp 1-del	Hp 2-del	Hp del–del	*P* value
N	377	2027	2834	133	306	10	–
Age (years)	60.71 ± 11.41	59.21 ± 12.21	59.86 ± 12.27	61.10 ± 11.53	58.52 ± 13.59	57.83 ± 9.17	0.0880
Male/female (n)	217/160	1110/917	1493/1341	80/53	173/133	5/5	0.1154
BMI (kg/m^2^)	24.71 ± 3.31	24.94 ± 5.52	24.69 ± 3.64	24.83 ± 3.26	24.67 ± 3.80	24.85 ± 1.87	0.5326
Diastolic blood pressure (mmHg)	80 (70, 86)	80 (75, 88)	80 (75, 88)	80 (70, 90)	80 (75, 85)	80 (70, 85)	0.1924
Systolic blood pressure (mmHg)	130 (120, 141)	130 (120, 140)	130 (120, 142)	130 (120, 145)	130 (120, 140)	120 (120, 140)	0.6499
Duration of diabetes (years)	7 (3, 12)	8 (3, 12)	8 (3, 13)	10 (4, 14)	9 (2, 13)	7 (3, 12)	0.4813
HbA1c (%) [mmol/mol]	8.8 (7.2, 10.5) [73 (55, 91)]	8.7 (7.3, 10.4) [72 (56, 90)]	8.6 (7.1, 10.4) [71 (54, 90)]	8.4 (6.9, 10.1) [68 (52, 87)]	8.8 (7.3, 10.4) [73 (56, 90)]	9.1 (6.9, 9.9) [76 (52, 85)]	0.6758
HDL-C (mmol/L)	1.07 (0.89, 1.29)	1.07 (0.90, 1.29)	1.09 (0.92, 1.30)	1.09 (0.91, 1.30)	1.10 (0.91, 1.27)	1.17 (1.09, 1.30)	0.0931
LDL-C (mmol/L)	2.86 (2.31, 3.41)	2.89 (2.31, 3.45)	2.97 (2.42, 3.58)	2.92 (2.34, 3.68)	3.08 (2.43, 3.81)	3.10 (2.33, 3.51)	< *0.0001*
Total cholesterol (mmol/L)	4.60 (3.87, 5.40)	4.60 (3.94, 5.30)	4.70 (4.10, 5.47)	4.67 (4.00, 5.50)	4.80 (4.18, 5.63)	4.82 (3.60, 5.30)	< *0.0001*
Triglycerides (mmol/L)	1.41 (0.98, 2.10)	1.41 (0.96, 2.13)	1.44 (1.00, 2.13)	1.49 (0.97, 1.88)	1.47 (1.00, 2.01)	1.17 (1.09, 1.36)	0.5292
Macroangiopathy disease	296 (78.51%)	1471 (72.57%)	2052 (72.41%)	105 (78.95%)	222 (72.55%)	7 (70.00%)	0.0793

Data are shown as n or mean ± standard deviation or median (interquartile range)

*Hp* haptoglobin, *BMI* body mass index, *HDL-C* high-density lipoprotein cholesterol, *LDL-C* low-density lipoprotein cholesterol

P values indicate the significance of differences among common Hp genotypes and clinical traits in subjects without the Hp del allele. *P* < 0.05 are shown in italics

### Association between common Hp genotypes and serum Hp levels

**Fig. 1:**
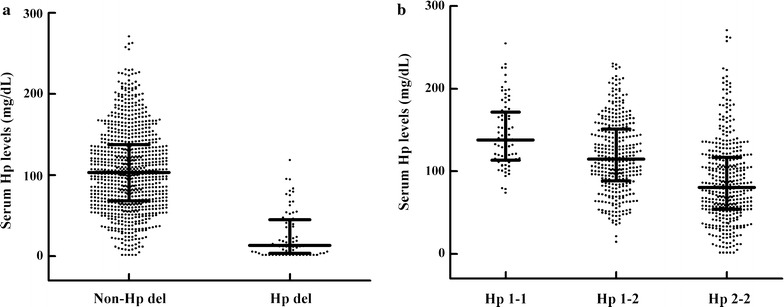
Serum haptoglobin (Hp) levels in 935 patients grouped by Hp genotype. The Hp levels are shown in dot plots; the median is indicated by the middle of the black solid line. The lower and upper quartiles are shown by the bottom and top of the black solid line, respectively. **a** Presents a comparison of serum Hp levels between non-Hp del (common Hp genotypes) and Hp del carriers, *P* = 2.61 × 10^−30^; **b** presents a comparison of the serum Hp levels among different common Hp genotypes (*P* = 3.55 × 10^−31^) based on a multiple linear regression analysis

The clinical characteristics of 935 patients grouped by Hp genotype are presented in Table 2. The common Hp genotypes were in Hardy–Weinberg equilibrium (*P* = 0.9661). Compared with the Hp del allele carriers, subjects with common Hp genotypes had significantly higher serum Hp levels (*P* = 2.61 × 10^−30^) (Fig. 1a). Further, after adjusting for age, sex, BMI, smoking, Hb level, and ALT, AST, albumin and hsCRP concentrations, common Hp genotypes were significantly and independently correlated with serum Hp levels (logarithmically transformed); the explained variance was 14.5% (Fig. 1b) (β_1_ ± SE = 0.1662 ± 0.014 for the Hp 1 allele, *P* = 3.55 × 10^−31^).

**Table 2:** Clinical characteristics of the 935 patients by Hp genotype

Variable	Hp 1-1	Hp 1-2	Hp 2-2	Hp 1-del	Hp 2-del	Hp del–del	*P* value
N	81	365	414	14	60	1	–
Age (year)	55.03 ± 11.39	53.16 ± 13.68	51.72 ± 14.58	56.21 ± 16.54	51.72 ± 13.94	52.36	0.1669
Male/female (n)	54/27	216/149	229/185	8/6	43/17	0/1	0.1403
BMI (kg/m^2^)	25.62 ± 3.53	25.36 ± 3.81	25.46 ± 4.31	26.31 ± 2.85	25.02 ± 4.13	24.84	0.9042
Diastolic blood pressure (mmHg)	80 (70, 90)	80 (75, 85)	80 (72, 85)	78 (74, 82)	80 (74, 84)	70	0.2114
Systolic blood pressure (mmHg)	130 (120, 142)	130 (120, 140)	130 (120, 140)	130 (120, 150)	125 (120, 140)	110	0.0520
Duration of diabetes (years)	10 (4, 14)	8 (4, 12)	8 (3, 13)	9 (5, 12)	10 (1, 14)	7	0.4326
HbA1c (%) [mmol/mol]	8.3 (6.8, 10.4) [67 (51, 90)]	8.3 (7.2, 10.1) [67 (55, 87)]	8.5 (7.1, 10.2) [69 (54, 88)]	8.1 (7.3, 9.5) [65 (56, 80)]	7.8 (6.9, 10.3) [62 (52, 89)]	6.0	0.9756
HDL-C (mmol/L)	0.96 (0.81, 1.20)	1.00 (0.88, 1.19)	1.00 (0.87, 1.20)	1.05 (0.93, 1.30)	1.08 (0.90, 1.19)	1.30	0.6729
LDL-C (mmol/L)	2.90 (2.20, 3.54)	2.73 (2.10, 3.28)	2.81 (2.23, 3.39)	2.94 (2.35, 3.48)	2.75 (2.01, 3.41)	2.70	0.2549
Total cholesterol (mmol/L)	4.68 (3.80, 5.70)	4.60 (4.00, 5.25)	4.72 (3.97, 5.40)	4.39 (4.20, 5.42)	4.61 (4.01, 5.36)	4.80	0.4200
Triglycerides (mmol/L)	1.40 (1.00, 1.94)	1.47 (0.98, 2.09)	1.47 (1.00, 2.17)	1.53 (1.08, 1.90)	1.47 (0.92, 2.20)	1.00	0.6222
ALT (IU/L)	18 (13, 28)	21 (14, 30)	21 (14, 32)	24 (15, 33)	19 (13, 31)	11	0.4981
AST (IU/L)	18 (15, 23)	18 (15, 24)	18 (15, 24)	21 (18, 33)	18 (13, 22)	14	0.3941
Albumin (g/L)	43 (41, 45)	43 (41, 45)	43 (41, 46)	45 (42, 48)	42 (41, 45)	41	0.6930
HsCRP (mg/L)	0.92 (0.47, 2.65)	0.99 (0.50, 2.23)	1.08 (0.48, 2.53)	1.69 (0.91, 6.13)	0.99 (0.46, 2.29)	0.17	0.5477
Haemoglobin (g/L)	139.14 ± 14.32	141.40 ± 15.43	139.04 ± 15.03	136.93 ± 11.09	140.66 ± 16.06	134	0.1324
Macroangiopathy disease	51 (62.96%)	201 (55.07%)	198 (47.83%)	11 (78.57%)	39 (65.00%)	0	*0.0044*

Data are shown as n or mean ± standard deviation or median (interquartile range)

*Hp* haptoglobin, *BMI* body mass index, *HDL-C* high-density lipoprotein cholesterol, *LDL-C* low-density lipoprotein cholesterol, *ALT* alanine aminotransferase, *AST* aspartate aminotransferase, *hsCRP* high sensitive C-reactive protein

*P* values indicate the significance of differences among common Hp genotypes and clinical traits in subjects without the Hp del allele. *P* values < 0.05 are shown in italic

### MR analysis to assess a causal effect of Hp on macroangiopathy

**Fig. 2:**
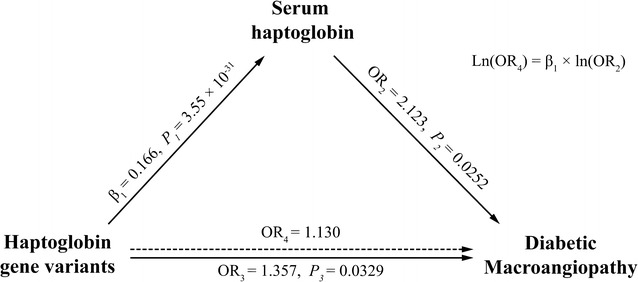
Framework of the Mendelian randomization analysis used in this study. The solid lines represent the observed associations in the three-way study: β_1_ for the effect size estimate of common variants of the Hp (haptoglobin) gene on serum Hp levels, OR_2_ for the association between Hp levels and diabetic macroangiopathy, and OR_3_ for the observed association between common variants of the Hp gene and diabetic macroangiopathy. The dashed line represents the predicted association between common variants of the Hp gene and diabetic macroangiopathy: OR_4_ was calculated as exp(β_1_ × log_e_ OR_2_). OR_3_ for the observed association and OR_4_ for the predicted association between common variants of the Hp gene and diabetic macroangiopathy in the current study were both larger than 1

The serum Hp levels in type 2 diabetes patients with and without macroangiopathy were significantly different (*P* = 8.83 × 10^−5^), with median (interquartile range) values of 109.34 (76.63, 143.26) mg/dL and 96.25 (58.68, 131.81) mg/dL, respectively. After adjusting for age, sex, BMI, blood pressure, duration of diabetes, HbA1c, LDL-C and TC, a significant correlation remained between serum Hp levels and macroangiopathy (OR_2_ = 2.123 [95% CI 1.098–4.102], *P* = 0.0252). The prevalence of macroangiopathy significantly differed among patients with different common Hp genotypes (*P* = 0.0044). We found that common Hp genotypes were significantly correlated with macroangiopathy after adjusting for age, sex, BMI, blood pressure, duration of diabetes, HbA1c, LDL-C and TC (OR_3_ = 1.357 [95% CI 1.025–1.798], *P* = 0.0329 for the Hp 1 allele). The MR framework for estimating the causal effects of Hp on macrovascular diseases in patients with type 2 diabetes in this study is described in Fig. 2. The directional trend of the observed and predicted correlation between common Hp genotypes and macroangiopathy were the same (OR_3_ and OR_4_ 1.357 and 1.130, respectively).

### Association between Hp and oxidative levels

**Fig. 3:**
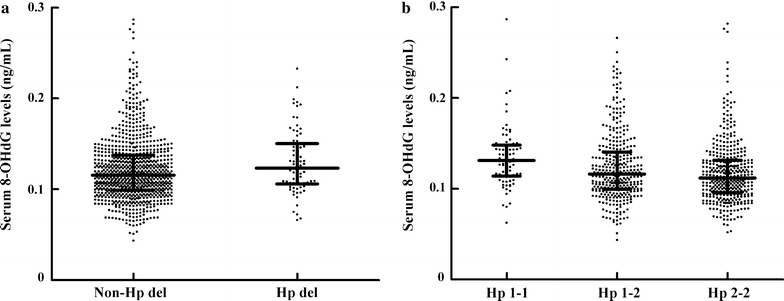
Serum 8-OHdG levels in 935 patients grouped by Hp genotype. *8-OHdG* 8-hydroxy-2′-deoxyguanosine, *Hp* haptoglobin. The 8-OHdG levels are shown in dot plots; the median is indicated by the middle of the black solid line. The lower and upper quartiles are shown by the bottom and top of the black solid line, respectively. **a** Presents a comparison of serum 8-OHdG levels between non-Hp del (common Hp genotypes) and Hp del carriers, *P* = 0.0548; **b** presents comparisons of serum 8-OHdG levels among common Hp genotypes, *P* = 0.0001, based on multiple linear regression analysis

A trend towards higher serum 8-OHdG levels was observed in patients with the Hp del allele (*P* = 0.0548) (Fig. 3a). Among those patients with common Hp genotypes, Hp genotype was significantly correlated with serum 8-OHdG level after adjusting for age, sex and BMI (Fig. 3b) (*P* = 0.0001). Additionally, there was a significant positive association between serum Hp levels and 8-OHdG levels in subjects with the common Hp genotypes after adjusting for age, sex, BMI, smoking, Hb level, and ALT, AST, albumin and hsCRP concentrations (*P* = 0.0084). Furthermore, serum 8-OHdG levels were compared among diabetic macroangiopathies patients in different classes (cerebral, carotid, coronary and lower limb atherosclerosis). Most of these patients also had another kind of atherosclerosis; therefore, the sample size in the subgroup with isolated cerebral or carotid arteriosclerosis is too small to achieve statistical power. The serum 8-OHdG levels were further compared between patients with isolated carotid atherosclerosis (n = 72) and lower limb arteriosclerosis (n = 123), and no significant difference was found (*P* = 0.0832).

## Discussion

The current study focuses on the causal role of serum Hp in the susceptibility to diabetic macroangiopathy based on an approach of MR analysis. As an IV, common Hp genotypes established their relationship with the susceptibility to macroangiopathy through their association with serum Hp levels. Considering the same directional trend of the predicted and observed associations between common Hp genotypes and diabetic macroangiopathy, we conclude that serum Hp is a risk factor for macroangiopathy in type 2 diabetes.

The role of Hp as an acute-phase protein in the early assessment of risk for CVDs has become a heavily discussed topic. A higher level of serum Hp has been observed in experimental diabetic rats during the early stage of diabetes [18]. In a previous cohort study, serum Hp level was significantly and independently correlated with the risk of cardiovascular death [19]. Another observational study revealed that serum Hp levels were significantly higher in patients with coronary artery disease [14]. However, whether serum Hp is a causal or concomitant factor of macroangiopathy in type 2 diabetes is unclear.

It is widely known that an RCT is recommended as the standard study design to assess the causality of a biomarker in human epidemiological studies [33]. Although an RCT exhibits many strengths, the high costs associated with the necessary time and material resources limit the applicability of RCT. Due to the interference of selection bias, confounding factors or reverse causation, it is hard to determine the causality of biomarkers in observational research. Thus, a rational alternative approach is needed to address the problems that arise in an observational study and to determine a causal relationship between risk factors and diseases.

Recently, vast improvements have been made in the exploration of MR studies in genetic epidemiology. With the discovery of multiple genetic variants by genome-wide association studies, it seems that MR studies have an even greater advantage by using genetic variants as a proxy for risk factors or modifiable exposures correlated with diseases. As described previously in the comparison of study design, the random allocation of an allele at meiosis in an MR study is similar to the random assignment of patients in an RCT, where the confounders are equal between groups [34]. By sharing many features with RCT, MR studies can determine whether the verified observational associations between risk factors and macroangiopathy are causal using related gene variants as an IV based on the laws of random and independent allele allocation. It is worth noting that several assumptions should be met as a key prerequisite before the utility of MR; therefore, an MR study adds to established study designs, such as RCT, rather than fully replacing them [35].

Considerable evidence has accumulated demonstrating that oxidative stress plays an important role in the pathophysiological mechanisms of CVDs [36]. A 10-year, follow-up, longitudinal study revealed that Hp 1-1 carriers had twice the risk for coronary heart disease-related mortality [37]. This finding prompted researchers to concentrate on CVDs in diabetes, considering the higher oxidative stress levels in diabetes, which might highlight the role of Hp. As an oxidation product of deoxyguanosine, 8-OHdG is used to determine the extent of DNA damage and evaluate oxidative stress levels in patients [38]. We observed that serum 8-OHdG levels were significantly associated with the common Hp genotypes and increased with the number of Hp 1 alleles, suggesting more severe oxidative stress and a higher risk of macroangiopathy in those subjects carrying this allele.

Furthermore, a higher serum Hp level was found in patients with the common genotype of Hp 1-1. We speculated that the synthesis of Hp protein was increased to make the antioxidant functions fully played due to the more severe oxidative stress in those patients. As a study recently reported, a higher Hp level was independently associated with poor overall survival in acute myocardial infarction patients [39]. It was hypothesized that the Hp level may reflect the severity of the oxidative stress and be a potential determinant of macrovascular disease risk. Additionally, serum Hp may play a more complex role in view of its interaction with other key proteins, which contributed to atherogenesis in diabetic macroangiopathy [40]. Thus, further functional studies are needed to reveal the potential role of serum Hp in linking oxidative stress and macroangiopathy in type 2 diabetes.

As reported previously, atherosclerosis leads to macroangiopathy and is responsible for the majority of deaths in patients with diabetes [41]. Clinical manifestations of atherosclerosis occur primarily in the macrovascular beds of coronary arteries, lower extremities, and carotid arteries. Due to the different pathogenesis of atherosclerosis in various arteries, the protein of Hp might play a different role in the proatherogenic progression. Most previous studies conducted with diabetic subjects found that Hp 2-2 was a genotypic risk factor for CVDs in diabetes [42, 43]. These studies used coronary artery disease as an endpoint, while we used a compound of macrovascular outcomes, including cerebral, carotid, coronary and lower limb atheroscleroses, in our analyses. After dividing macroangiopathy into four classes based on the outcomes mentioned above, Hp 1-1 was found to be associated with a significantly higher risk of carotid atherosclerosis.

Carotid atherosclerosis was reported to be associated with an increased risk of stroke and poorer cognitive performance in the elderly [44]. In addition, patients with the Hp 1-1 genotype were identified to have an increased risk of stroke in a longitudinal study of type 1 diabetes and poorer cognitive function in elderly individuals with type 2 diabetes [45, 46]. The influence of Hp genotypes on stroke might be partly attributed to their association with carotid atherosclerosis, which appears to have a different aetiology than that of coronary artery disease. Dysregulations of multiple cell types, including endothelium, smooth muscle cells, and platelets, played a key role in the proatherogenic progression [41]. Functional research is needed to reveal the mechanism underlying this difference.

Serum Hp levels are genetically determined, mainly by the common Hp genotypes [47]. However, the concentration of serum Hp might be affected by other factors and by multi-diseases status. In previous reports, age, sex, smoking, and plasma Hb levels were shown to be associated with the serum level of Hp [10]. As a marker of acute phase response, the synthesis of Hp is increased during acute inflammation [48]. Additionally, serum Hp concentrations decrease in patients with haemolysis, ineffective erythropoiesis, late pregnancy, malnutrition and chronic liver disease [10]. In this study, patients with haemolysis, ineffective erythropoiesis and pregnancy were excluded from this study. Furthermore, we have measured the levels of ALT and AST to evaluate liver function [23], serum albumin to assess nutritional status [24] and serum hsCRP as a blood biomarker of acute inflammation screening [49]. In the analysis of serum Hp levels, the association between Hp levels and the common Hp genotypes remained significant after adjusting for all these confounding factors. For a genetic variant to be considered a valid IV according to Mendel’s laws, there must exist robust evidence of a true association between the genetic variant and exposure of interest [21]. As the coding gene of Hp protein, the common Hp genotypes established a reliable association with serum Hp levels independent of other confounders in the current study and in previous reports [14, 15, 39].

The Hp del, an allelic deletion larger than 20 kb, is associated with extremely low levels of serum Hp. Homozygosity for Hp del was first reported in Japan in an individual with anhaptoglobinemia. Furthermore, the same study found seven heterozygous Hp/Hp del individuals in three families with hypohaptoglobinemia [9]. In our study, the serum Hp levels in Hp/Hp del patients were significantly lower than in Hp/Hp carriers, consistent with the results previously reported in East and Southeast Asian populations [10]. Recently, it was reported that a splice donor founder mutation on the Hp 1 allele is correlated with a lower level of serum Hp and a higher risk of CVD [50]. In this study, we observed that subjects with Hp 2-2 had lower serum Hp concentrations than those with the other Hp genotypes, similar to findings in European and Japanese populations [10]. In addition, we observed a trend towards higher 8-OHdG levels in individuals carrying the Hp del allele, which might result from weaker antioxidant activity caused by anhaptoglobinemia in these patients.

Strengths of the present study include the validated diagnosis of macroangiopathy in a large sample of patients with type 2 diabetes. To the best of our knowledge, this is the first study to use MR analysis to investigate the causal relationship between serum Hp levels and macroangiopathy in type 2 diabetes. However, this study has certain limitations. First, we cannot exclude the possibility of selection and population bias in our study, because the MR analysis was performed with only 935 patients from the total population. A larger number of subjects is required to fully investigate the effect of serum Hp levels on macroangiopathy. Second, although a significant relationship between serum haptoglobin levels and macroangiopathy was observed, the value of OR was not as high as expected. This finding shows that the causal role for serum Hp in promoting macroangiopathy might be limited, compared to other established risk factors, such as hyperglycaemia, hypertension and dyslipidaemia. Therefore, careful consideration should be paid to determining whether serum Hp should be a biomarker of clinical diagnosis and therapeutic targets. Moreover, functional explorations of the role of Hp in vivo and in vitro are warranted to reveal the underlying mechanisms and interactions between serum Hp and macroangiopathy in patients with type 2 diabetes. Third, several confounding lifestyle factors that might influence Hp or 8-OHdG levels were not considered. Finally, although we used common variants of the Hp gene as an IV to assess the causality of Hp levels in macroangiopathy, the role of pleiotropy cannot be completely excluded.

## Conclusions

In brief, our study provides new support for the causal role of serum Hp in macroangiopathy using the MR analysis method with common variants of the Hp gene as an IV in Chinese type 2 diabetes patients. Moreover, we suppose that the serum Hp level may be associated with the severity of the oxidative stress in the process of diabetic macroangiopathy and further functional investigations are required to uncover the underlying mechanisms. This finding gives people a new perspective on prevention strategies by highlighting the role of circulating serum Hp levels in the development of macroangiopathy in type 2 diabetes.

## Additional file

**Additional file 1: Table S1.** Association between common Hp genotypes and clinical traits. **Table S2.** Various atheroscleroses grouped by common Hp genotypes.

## Abbreviations

Hp: haptoglobin
Hb: haemoglobin
CVDs: cardiovascular diseases
MR: mendelian randomization
RCT: randomized controlled trial
8-OHdG: 8-hydroxy-2′-deoxyguanosine
BMI: body mass index
TC: total cholesterol
TG: triglycerides
HDL-C: high-density lipoprotein cholesterol
LDL-C: low-density lipoprotein cholesterol
ALT: alanine aminotransferase
AST: aspartate aminotransferase
hsCRP: high sensitive C-reactive protein
ELISA: enzyme-linked immunosorbent assay
IV: instrumental variable
OR: odds ratio
CI: confidence interval
