# Supplementary material for: Mendelian randomization analysis to assess a causal effect of haptoglobin on macroangiopathy in Chinese type 2 diabetes patients
Source: Cardiovasc Diabetol. 2018 Jan 16;17:14. doi: 10.1186/s12933-018-0662-7 (PMC5769522; doi:10.1186/s12933-018-0662-7)
Supplement: Supplementary file 1 — Additional file 1: Table S1. Association between common Hp genotypes and clinical traits. Table S2. Various atheroscleroses grouped by common Hp genotypes. [file 12933_2018_662_MOESM1_ESM.docx]

**Supplemental** **Table S1**

**Association between common Hp genotypes and clinical traits**

| Variable | β for minor allele (Hp 1) | SE | P value |
| --- | --- | --- | --- |
| Duration of diabetes (years) | -0.0201 | 0.0150 | 0.1801 |
| HbA1c | 0.0029 | 0.0024 | 0.2346 |
| HDL-C | -0.0044 | 0.0026 | 0.0832 |
| LDL-C | -0.0135 | 0.0031 | **1.59 × 10^-5^** |
| Total cholesterol | -0.0099 | 0.0023 | **1.45 × 10^-5^** |
| Triglycerides | -0.0048 | 0.0059 | 0.4163 |

All skewed quantitative traits were logarithmically transformed for analysis with adjustment for age, sex, body mass index, diastolic and systolic blood pressures. Hp: haptoglobin; HDL-C: high-density lipoprotein cholesterol; LDL-C: low-density lipoprotein cholesterol; *P* values < 0.05 are shown in bold.

**Supplemental Table S2**

**Various atheroscleroses grouped by common Hp genotypes**

| **Variable** | **Hp 1-1** | **Hp 1-2** | **Hp 2-2** | **OR** | **95% CI** | ***P* value** |
| --- | --- | --- | --- | --- | --- | --- |
| Type 2 diabetes without macroangiopathy disease | 81 | 556 | 782 | -- | -- | -- |
| Cerebral atherosclerosis | 42 (7.22%) | 239 (41.07%) | 301 (51.71%) | 1.083 | 0.854, 1.374 | 0.5095 |
| Carotid atherosclerosis | 194 (7.88%) | 938 (38.10%) | 1330 (54.02%) | 1.183 | 1.027, 1.363 | **0.0202** |
| Coronary atherosclerosis | 2 (4.76%) | 10 (23.81%) | 30 (71.43%) | 0.566 | 0.273, 1.175 | 0.1266 |
| Lower limb atherosclerosis | 221 (7.33%) | 1173 (38.93%) | 1619 (53.74%) | 1.096 | 0.957, 1.255 | 0.1847 |

Data are shown as n or n (proportion). Hp: haptoglobin; OR: odds ratio; CI: confidence interval. Cerebral atherosclerosis includes stroke and intracerebral hemorrhage; coronary atherosclerosis includes myocardial infarction. The association between common Hp genotypes and each atherosclerosis was tested using multivariable logistic regression analysis after adjusting for age, sex, body mass index, blood pressure, duration of diabetes, HbA1c, low-density lipoprotein cholesterol and total cholesterol , and the OR with 95% CI were presented. *P* values < 0.05 are shown in bold.
